# Serum 1,5‐Anhydroglucitol Identifies Residual Mortality Risk Beyond Time in Range in Type 2 Diabetes: A Cohort Study

**DOI:** 10.1111/1753-0407.70260

**Published:** 2026-08-04

**Authors:** Jiaying Ni, Lei Chen, Hang Su, Jingyi Guo, Chunfang Wang, Yufei Wang, Jingyi Lu, Jian Zhou, Tian Xia, Feng Jiang

**Affiliations:** ^1^ Department of Endocrinology and Metabolism, Shanghai Sixth People's Hospital Affiliated to Shanghai Jiao Tong University School of Medicine, Shanghai Clinical Center for Diabetes Shanghai Diabetes Institute, Shanghai Key Laboratory of Diabetes Mellitus Shanghai China; ^2^ Vital Statistical Department Institute of Health Information, Shanghai Municipal Center for Disease Control and Prevention Shanghai China; ^3^ Department of Gerontology Shanghai Sixth People's Hospital Affiliated to Shanghai Jiao Tong University School of Medicine Shanghai China; ^4^ Clinical Research Center Shanghai Sixth People's Hospital Affiliated to Shanghai Jiao Tong University School of Medicine Shanghai China

**Keywords:** 1,5‐anhydroglucitol, all‐cause mortality, continuous glucose monitoring, time in range, type 2 diabetes

## Abstract

**Background:**

The combined prognostic value of continuous glucose monitoring (CGM) metrics and circulating glucose biomarkers for predicting mortality in type 2 diabetes has not been fully established, particularly regarding residual risk in patients achieving glycemic targets.

**Methods:**

This cohort study included 3677 patients with type 2 diabetes for a median of 7.4 years follow‐up. Cox proportional hazards models evaluated the associations of baseline CGM‐derived time in range (TIR) (target 70%) and serum 1,5‐anhydroglucitol (1,5‐AG) (threshold 6.0 μg/mL) with all‐cause and cardiovascular mortality in the overall population. We further examined the relationship between 1,5‐AG and mortality within TIR subgroups and compared its performance with metrics of glycemic variability derived from CGM.

**Results:**

During follow‐up, 522 all‐cause deaths and 181 cardiovascular deaths occurred. TIR and 1,5‐AG were moderately correlated and independently predicted mortality, with concurrent low TIR (≤ 70%) and low 1,5‐AG (< 6.0 μg/mL) yielding the highest risk (hazard ratio [HR] 1.82, 95% CI 1.40–2.37). In stratified analyses, reduced 1,5‐AG was significantly associated with increased mortality risk in patients with TIR > 70% (HR 1.69, 95% CI 1.25–2.28), whereas no significant association was observed in those with TIR ≤ 70%. Adding 1,5‐AG improved traditional risk prediction in the former subgroup. Compared with 1,5‐AG, CGM‐derived glycemic variability indices such as mean amplitude of glycemic excursions, coefficient of variation, and standard deviation of glucose showed no significant association with mortality.

**Conclusions:**

TIR and serum 1,5‐AG offer independent and complementary value, with low 1,5‐AG identifying residual mortality risk despite achieving TIR targets.

## Highlights

- Serum 1,5‐anhydroglucitol (1,5‐AG) may capture glycemic variability beyond the scope of time in range (TIR) derived from continuous glucose monitoring.
- TIR and serum 1,5‐AG have independent, complementary prognostic value in type 2 diabetes.
- Low serum 1,5‐AG levels (< 6.0 μg/mL) identify residual mortality risk in participants with TIR > 70%.

## Graphical abstract

Serum 1,5‐anhydroglucitol (1,5‐AG) and time in range (TIR) derived from continuous glucose monitoring have independent, complementary prognostic value in type 2 diabetes. Serum 1,5‐AG may capture glycemic variability beyond the scope of TIR and identifies residual mortality risk in participants with TIR > 70%.

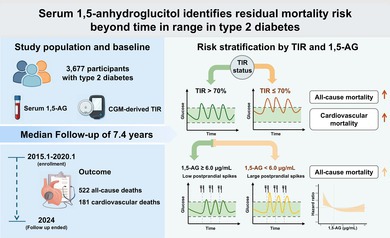

## 1 Introduction

The rising global burden of diabetes emphasizes the critical impact of glycemic control on cardiovascular outcomes and all‐cause mortality [1]. While glycated hemoglobin A1c (HbA1c) remains the gold standard for assessment, it mainly represents mean glucose exposure and provides limited information on postprandial hyperglycemia and glycemic variability [2]. Continuous glucose monitoring (CGM) provides detailed glucose data, supporting the incorporation of time in range (TIR) into international recommendations as an important indicator of glucose control [3, 4, 5]. Our previous studies have confirmed that lower TIR is related to elevated risks of macrovascular events and death in type 2 diabetes [6, 7]. However, TIR correlates strongly (*r* > 0.9) with hyperglycemia exposure, including time above range (TAR) and mean sensor glucose (MSG) [8]. Furthermore, the relationship between TIR and MSG is modified by glycemic variability [9]. Consequently, TIR primarily reflects hyperglycemic exposure and may not distinguish between patients who achieve similar TIR targets but experience varying degrees of glycemic variability.

Glycemic variability is an established cardiovascular risk factor [10, 11, 12, 13]. Mechanistic studies indicate that acute glucose fluctuations induce oxidative stress, subsequently leading to endothelial dysfunction and accelerating vascular pathology [14, 15, 16, 17]. Serum 1,5‐anhydroglucitol (1,5‐AG) reflects short‐term glycemic status [18]. When plasma glucose exceeds the renal threshold (~10.0 mmol/L), excess glucose in the renal tubules competitively inhibits 1,5‐AG reabsorption, thereby increasing its urinary excretion and lowering its circulating concentration [19]. Its 1–2 week monitoring window aligns with the typical CGM duration, supporting their combined clinical use [20]. Prior evidence indicates that 1,5‐AG provides a more sensitive assessment of glycemic variability than HbA1c in individuals with mild dysglycemia [21].

Although TIR and serum 1,5‐AG have shown prognostic relevance individually [7, 22], their combined utility in predicting long‐term outcomes remains underexplored. Therefore, this study aimed to determine whether TIR and serum 1,5‐AG independently and jointly contribute to the prediction of all‐cause and cardiovascular mortality in type 2 diabetes.

## 2 Methods

### 2.1 Study Design and Population

Data were obtained from the INDices of contInuous Glucose monitoring and adverse Outcomes of diabetes (INDIGO) study, which began in 2005 at Shanghai Sixth People's Hospital Affiliated to Shanghai Jiao Tong University School of Medicine. To ensure the availability of 1,5‐AG measurements, this analysis focuses on a distinct dataset collected between January 2015 and January 2020, which differs from previous publications based on the earlier 2005 to 2015 cohort [7, 23]. The study population comprised hospitalized adults with type 2 diabetes who underwent complications screening and glycemic assessment at a tertiary center. Eligible participants were required to be aged ≥ 18 years with type 2 diabetes, have available 1,5‐AG and CGM measurements, hold permanent residency in Shanghai, and maintain a stable antidiabetic regimen during the 3 months preceding admission. Patients with other diabetes types, diabetic ketoacidosis at admission, or sodium‐glucose cotransporter 2 (SGLT2) inhibitor use were excluded.

The study protocol was reviewed and approved by the Ethics Committee of Shanghai Sixth People's Hospital Affiliated to Shanghai Jiao Tong University School of Medicine and was implemented in accordance with the Declaration of Helsinki. All participants gave written informed consent before being enrolled.

### 2.2 Clinical and Laboratory Assessments

Demographic characteristics were retrieved from electronic medical records. Anthropometric measurements were conducted by trained nurses. Laboratory measurements, including HbA1c, serum 1,5‐AG, fasting plasma glucose, and lipid profiles, were performed using fasting venous blood samples [22] and estimated glomerular filtration rate (eGFR) was derived from serum creatinine using the Chronic Kidney Disease Epidemiology Collaboration equation [24]. Albuminuria and diabetic retinopathy were defined according to previously established protocols [25].

### 2.3 CGM Parameters Calculation

Retrospective CGM was performed using the iPro2 or CGMS GOLD systems (Medtronic). Sensors were inserted upon hospital admission and removed after approximately 72 h. Interstitial glucose was recorded at 5‐min intervals and calibrated at least semi‐daily using capillary blood glucose (SureStep, LifeScan). To ensure data robustness and minimize day‐to‐day fluctuations, CGM metrics were calculated by averaging daily values across the full duration of valid monitoring. We calculated range‐based indices, MSG and three variability indices: standard deviation (SD), coefficient of variation (CV), and mean amplitude of glycemic excursions (MAGE). Throughout the monitoring period, participants followed a standardized isocaloric diet (25 kcal/kg/day) comprising 55% carbohydrate, 17% protein, and 28% fat.

### 2.4 Study Outcomes

Information on all‐cause and cause‐specific death was obtained from the Shanghai Center for Disease Control and Prevention registry through linkage using unique personal identification numbers. All participants included in the analytic cohort were successfully linked to the registry. Cardiovascular deaths were defined by International Classification of Diseases, 10th version (ICD‐10) codes I00–I99. The underreporting rate of death events has been estimated at 0.7‰ [7]. The main outcomes were death from any cause and cardiovascular causes, tracked until death or the censoring date of December 31, 2024.

### 2.5 Statistical Analysis

Analyses were implemented in R version 4.2.2 through RStudio (Posit PBC, Boston, MA, USA). Data distribution was first assessed for continuous variables, which are presented as mean ± SD or median (interquartile range [IQR]), as appropriate; categorical variables are summarized as *n* (%). Group comparisons were performed using one‐way ANOVA, Kruskal–Wallis *H* tests, or *χ* ^2^ tests. Correlations were quantified using Pearson's or Spearman's coefficients according to distributional characteristics. A two‐sided *p* < 0.05 was considered statistically significant. We defined a binary 1,5‐AG variable using the validated 6.0 μg/mL cutoff, which identifies recent peak glucose exceeding ~11.1 mmol/L [26]. TIR was categorized based on the standard clinical target of > 70% [5]. For joint‐risk assessment, four TIR/1,5‐AG categories were created, with TIR > 70% and 1,5‐AG ≥ 6.0 μg/mL as the reference.

Separate Cox proportional hazards models were fitted for TIR and 1,5‐AG to estimate their associations with all‐cause and cardiovascular mortality, with effect estimates reported as hazard ratios (HRs) and 95% confidence intervals (CIs). Covariates were selected a priori based on previous studies and clinical relevance [7, 22, 26]. Model 1 included only age and sex. Model 2, the primary analytical model, additionally included diabetes duration, body mass index (BMI), systolic blood pressure, lipid profile, smoking status, eGFR, history of cancer or cardiovascular disease, and medication use. Multicollinearity was assessed using variance inflation factors, all of which were below 2. Sensitivity analyses were conducted based on Model 2 and included the following: additional adjustment for HbA1c, fasting plasma glucose, CGM‐derived TAR, and time below range (TBR) in separate models; further adjustment for baseline diabetic complications among participants with available urine albumin and fundus examination data; and repetition of the analyses after excluding patients with eGFR < 30 mL/min/1.73 m^2^.

In analyses stratified by TIR status (> 70% or ≤ 70%), the predefined 6.0 μg/mL threshold was used for categorical 1,5‐AG analyses, whereas restricted cubic splines (RCS) with knots at the 10th, 50th, and 90th percentiles were used to assess continuous associations with mortality. As a sensitivity analysis within each TIR strata, serum 1,5‐AG was additionally categorized according to the median value to assess the robustness of the associations. The incremental prognostic value of adding 1,5‐AG categories to the established multivariable model (Model 2) was quantified using the C‐statistics, continuous net reclassification improvement (NRI), and integrated discrimination improvement (IDI) at 3, 5, and 7 years. Subgroup analyses were conducted across various baseline characteristics. Interactions between 1,5‐AG and relevant covariates were evaluated using likelihood ratio tests. Furthermore, we assessed the correlation of 1,5‐AG with CGM‐derived glycemic indices (MAGE, CV, SD) and compared their respective associations with risk of mortality in total and TIR > 70%. For each variability metric, values in the highest tertile were categorized as high‐risk status [27, 28].

## 3 Results

### 3.1 Baseline Clinical Characteristics of the Participants

Among the 3677 participants, four categories were defined using the combined status of TIR and 1,5‐AG (Figure S1, Table 1). Of these, 800 (21.8%) individuals had high TIR (> 70%) and high 1,5‐AG (≥ 6.0 μg/mL), whereas 1695 (46.1%) were classified into the group characterized by both low TIR (≤ 70%) and low 1,5‐AG (< 6.0 μg/mL). Males accounted for 58.0% of the study population. The cohort had an age of 62.0 years (IQR, 55.0–68.0) and diabetes duration of 11.0 years (IQR, 6.0–17.0). For glycemic indicators, 1,5‐AG was 3.3 μg/mL (IQR, 1.7–6.8), while TIR was 68.0% (IQR, 50.0–85.0).

**TABLE 1:** Characteristics of the study population.

	Total	Low TIR/low 1,5‐AG	Low TIR/high 1,5‐AG	High TIR/low 1,5‐AG	High TIR/high 1,5‐AG	*p*
Participants, *n*	3677	1695	283	899	800	
Sex, *n* (%)						< 0.001
Male	2133 (58.0%)	1025 (60.5%)	160 (56.5%)	536 (59.6%)	412 (51.5%)	
Female	1544 (42.0%)	670 (39.5%)	123 (43.5%)	363 (40.4%)	388 (48.5%)	
Age, years	62.0 (55.0, 68.0)	62.0 (55.0, 68.5)	66.0 (60.0, 72.0)	60.0 (53.0, 67.0)	62.0 (56.0, 69.0)	< 0.001
Diabetes duration, years	11.0 (6.0, 17.0)	12.0 (7.0, 19.0)	14.0 (9.0, 20.0)	10.0 (5.0, 15.0)	10.0 (5.0, 15.2)	< 0.001
Systolic blood pressure, mmHg	130.0 (120.0, 140.0)	130.0 (120.0, 144.0)	133.0 (120.0, 144.0)	130.0 (120.0, 140.0)	130.0 (120.0, 140.0)	< 0.001
Diastolic blood pressure, mmHg	80.0 (70.0, 85.0)	80.0 (70.0, 85.0)	80.0 (70.0, 82.0)	80.0 (70.0, 85.0)	80.0 (70.0, 84.0)	< 0.001
Body mass index, kg/m^2^	24.9 (22.8, 27.5)	24.8 (22.7, 27.3)	24.5 (22.3, 26.9)	25.2 (23.0, 27.7)	25.0 (22.7, 27.8)	0.006
eGFR, mL/min/1.73 m^2^	95.5 (84.2, 103.9)	96.5 (85.6, 105.6)	90.8 (74.9, 99.2)	97.0 (85.5, 106.1)	92.8 (81.0, 100.4)	< 0.001
Total cholesterol, mmol/L	4.6 (3.8, 5.3)	4.7 (3.9, 5.5)	4.5 (3.7, 5.1)	4.6 (3.8, 5.2)	4.4 (3.6, 5.1)	< 0.001
Triglycerides, mmol/L	1.4 (1.0, 2.1)	1.5 (1.0, 2.2)	1.4 (1.0, 2.0)	1.4 (1.0, 2.1)	1.4 (1.0, 2.0)	0.008
HDL cholesterol, mmol/L	1.0 (0.9, 1.2)	1.0 (0.9, 1.2)	1.0 (0.9, 1.3)	1.0 (0.9, 1.2)	1.0 (0.9, 1.2)	0.007
LDL cholesterol, mmol/L	2.7 (2.1, 3.4)	2.8 (2.2, 3.5)	2.7 (2.0, 3.2)	2.7 (2.1, 3.4)	2.5 (1.9, 3.1)	< 0.001
HbA1c, %	8.5 (7.3, 10.0)	9.6 (8.6, 10.8)	7.7 (7.0, 8.3)	8.5 (7.6, 9.9)	6.8 (6.3, 7.3)	< 0.001
1,5‐AG, μg/mL	3.3 (1.7, 6.8)	2.0 (1.3, 3.1)	8.3 (7.0, 11.4)	2.9 (1.7, 4.2)	10.6 (7.9, 15.6)	< 0.001
Fasting plasma glucose, mmol/L	7.5 (6.2, 9.2)	8.6 (7.0, 10.6)	7.5 (6.2, 8.7)	7.0 (6.0, 8.4)	6.3 (5.5, 7.3)	< 0.001
Current smoker, *n* (%)	943 (25.6%)	467 (27.6%)	55 (19.4%)	250 (27.8%)	171 (21.4%)	< 0.001
History of cancer, *n* (%)	115 (3.1%)	41 (2.4%)	11 (3.9%)	28 (3.1%)	35 (4.4%)	0.059
History of CVDs, *n* (%)	954 (25.9%)	456 (26.9%)	102 (36.0%)	194 (21.6%)	202 (25.2%)	< 0.001
Oral diabetic agents, *n* (%)	2648 (72.0%)	1169 (69.0%)	202 (71.4%)	650 (72.3%)	627 (78.4%)	< 0.001
Insulin, *n* (%)	1745 (47.5%)	927 (54.7%)	155 (54.8%)	415 (46.2%)	248 (31.0%)	< 0.001
Anti‐hypertension agents, *n* (%)	2048 (55.7%)	932 (55.0%)	164 (58.0%)	491 (54.6%)	461 (57.6%)	0.464
Lipid‐lowering agents, *n* (%)	2491 (67.7%)	1181 (69.7%)	193 (68.2%)	593 (66.0%)	524 (65.5%)	0.108
Antiplatelet drugs, *n* (%)	1582 (43.0%)	761 (44.9%)	131 (46.3%)	348 (38.7%)	342 (42.8%)	0.015
CGM metrics						
Mean sensor glucose, mmol/L	9.0 (7.9, 10.2)	10.2 (9.6, 11.3)	9.6 (9.0, 10.2)	8.1 (7.4, 8.6)	7.6 (7.0, 8.2)	< 0.001
SD of sensor glucose, mmol/L	2.3 (1.7, 2.9)	2.8 (2.2, 3.4)	2.6 (2.2, 3.2)	1.9 (1.4, 2.3)	1.7 (1.3, 2.1)	< 0.001
CV, %	24.8 (19.3, 30.8)	26.6 (21.1, 33.1)	27.8 (22.3, 34.3)	23.3 (18.0, 29.3)	22.0 (17.0, 26.9)	< 0.001
MAGE, mmol/L	5.1 (3.6, 6.8)	6.1 (4.6, 7.8)	5.9 (4.8, 7.7)	4.2 (2.9, 5.7)	3.8 (2.8, 5.0)	< 0.001
TAR, %	30.0 (13.0, 48.0)	48.0 (38.0, 65.0)	39.0 (32.0, 48.5)	16.0 (7.0, 22.0)	9.0 (3.0, 16.0)	< 0.001
TIR, %	68.0 (50.0, 85.0)	51.0 (35.0, 61.0)	59.0 (49.5, 66.0)	83.0 (76.0, 91.0)	89.0 (82.0, 95.0)	< 0.001
TBR, %	0.0 (0.0, 1.0)	0.0 (0.0, 0.0)	0.0 (0.0, 2.5)	0.0 (0.0, 1.0)	0.0 (0.0, 2.0)	< 0.001

*Note:* Data shown are median (interquartile range) or number (percentage) unless otherwise indicated. Low TIR was defined as TIR ≤ 70%, and high TIR was defined as TIR > 70%. Low 1,5‐AG was defined as 1,5‐AG < 6.0 μg/mL, and high 1,5‐AG was defined as 1,5‐AG ≥ 6.0 μg/mL.

Abbreviations: 1,5‐AG, 1,5‐anhydroglucitol; CGM, continuous glucose monitoring; CV, coefficient of variation; CVD, cardiovascular disease; eGFR, estimated glomerular filtration rate; HDL, high‐density lipoprotein; HbA1c, glycated hemoglobin A1c; LDL, low‐density lipoprotein; MAGE, mean amplitude of glycemic excursions; SD, standard deviation; TAR, time above range (> 10.0 mmol/L); TBR, time below range (< 3.9 mmol/L); TIR, time in range (3.9–10.0 mmol/L).

Patients with low TIR and low 1,5‐AG were predominantly male and exhibited poorer control of lipid profiles (all *p* < 0.05). Glycemic parameters varied across the four groups (all *p* < 0.001). HbA1c and CGM‐derived metrics (MSG, SD, MAGE, TAR) were highest in individuals with both low TIR and low 1,5‐AG, and lowest in those with high TIR and high 1,5‐AG. Regarding therapy, those with high TIR and high 1,5‐AG were treated more frequently with oral glucose‐lowering drugs and less frequently with insulin (all *p* < 0.001), although the proportions of patients receiving antihypertensive and lipid‐lowering agents were similar across all groups (all *p* > 0.05).

### 3.2 Associations of TIR, 1,5‐AG, and Joint Categories With Mortality

**FIGURE 1:**
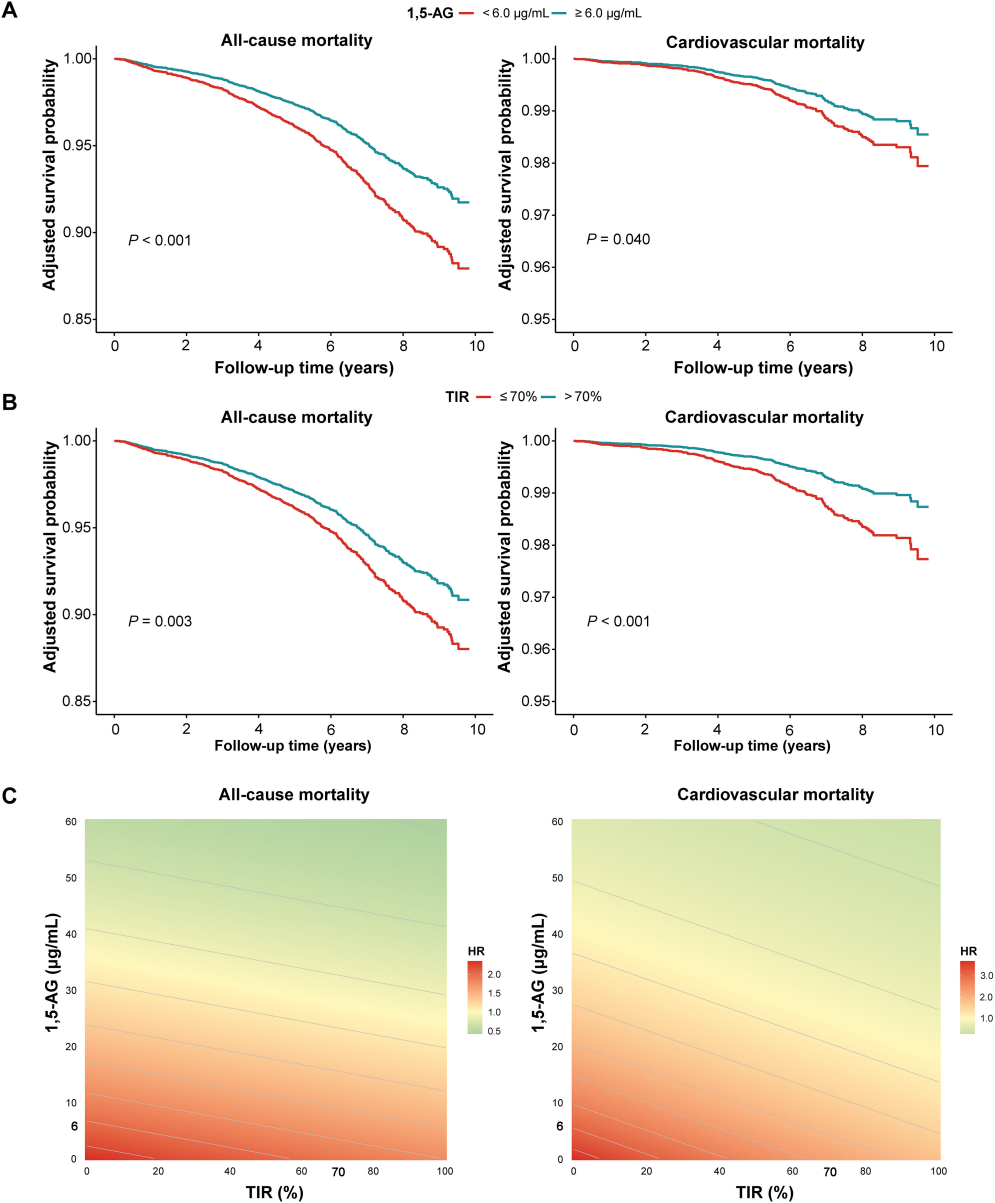
Adjusted mortality risk according to serum 1,5‐AG and TIR. Panel A: Cox‐adjusted survival curves for all‐cause mortality and cardiovascular mortality stratified by serum 1,5‐AG (< 6.0 vs. ≥ 6.0 μg/mL). Panel B: Cox‐adjusted survival curves for all‐cause mortality and cardiovascular mortality stratified by TIR (≤ 70% vs. > 70%). *p‐*Values indicate between‐group differences derived from Cox proportional hazards models. Panel C: Adjusted relative hazards of all‐cause mortality and cardiovascular mortality across the joint distribution of serum 1,5‐AG and TIR. All analyses were adjusted for age, sex, diabetes duration, body mass index, systolic blood pressure, triglycerides, high‐density lipoprotein cholesterol, low‐density lipoprotein cholesterol, current smoking status, estimated glomerular filtration rate, history of cancer, history of cardiovascular disease, and the use of insulin, oral hypoglycemic drugs, antihypertensive drugs, lipid‐lowering drugs, and antiplatelet drugs. 1,5‐AG, 1,5‐anhydroglucitol; HR, hazard ratio; TIR, time in range (3.9–10.0 mmol/L).

After a median follow‐up of 7.4 years, 522 participants had died, with cardiovascular causes accounting for 181 deaths. Adjusted survival curves demonstrated distinct outcome patterns across the individual TIR and 1,5‐AG categories (all *p* < 0.05, Figure 1A,B). In the corresponding multivariable Cox regression (Model 2), low 1,5‐AG (< 6.0 μg/mL) remained independently related to higher risk of all‐cause (HR 1.49, 95% CI 1.22–1.82) and cardiovascular mortality (HR 1.42, 95% CI 1.02–1.99; Table S1). A TIR ≤ 70% was likewise linked to elevated mortality risk after adjustment, corresponding to HRs of 1.33 (95% CI 1.10–1.60) for all‐cause and 1.80 (95% CI 1.29–2.51) for cardiovascular mortality (Table S2).

Although TIR and 1,5‐AG were moderately correlated (Spearman *r* = 0.475, *p* < 0.05), no significant interaction between TIR and 1,5‐AG was observed in relation to all‐cause mortality (*p* for interaction = 0.703) or cardiovascular mortality (*p* for interaction = 0.973). As illustrated in the two‐dimensional risk heatmap (Figure 1C), the lowest mortality risks clustered in the region corresponding to high TIR and high 1,5‐AG. Notably, risk remained elevated in the presence of low 1,5‐AG, even when TIR was maintained at optimal levels. In joint analyses, compared with the reference group (TIR > 70% and 1,5‐AG ≥ 6.0 μg/mL), individuals with combined low values exhibited the highest risks, with HRs of 1.82 (95% CI 1.40–2.37) for all‐cause and 2.09 (95% CI 1.33–3.27) for cardiovascular mortality (Table 2). These associations remained generally consistent across sensitivity analyses, including additional adjustment for glycemic metrics, albuminuria, and diabetic retinopathy and exclusion of patients with eGFR < 30 mL/min/1.73 m^2^ (Tables S3–S5).

**TABLE 2:** Joint associations of TIR and 1,5‐AG with all‐cause and cardiovascular mortality.

	Group			
	Low TIR/low 1,5‐AG	Low TIR/high 1,5‐AG	High TIR/low 1,5‐AG	High TIR/high 1,5‐AG
All‐cause mortality				
Number of participants	1695	283	899	800
Number of deaths	275	57	114	76
Person‐years	11944.03	1993.37	6473.94	5993.34
Incidence rate (per 1000 person‐years)	23.02	28.59	17.61	12.68
HRs (95% CIs)				
Unadjusted	1.86 (1.44–2.40)***	2.31 (1.64–3.26)***	1.42 (1.06–1.90)*	1.00
Model 1	1.81 (1.41–2.34)***	1.71 (1.22–2.42)**	1.65 (1.24–2.21)***	1.00
Model 2	1.82 (1.40–2.37)***	1.58 (1.11–2.24)*	1.69 (1.26–2.27)***	1.00
Cardiovascular mortality				
Number of deaths	105	23	28	25
Incidence rate (per 1000 person‐years)	8.79	11.54	4.33	4.17
HRs (95% CIs)				
Unadjusted	2.17 (1.40–3.35)***	2.85 (1.62–5.02)***	1.06 (0.62–1.82)	1.00
Model 1	2.12 (1.37–3.28)***	2.01 (1.14–3.55)*	1.28 (0.75–2.20)	1.00
Model 2	2.09 (1.33–3.27)**	1.80 (1.01–3.21)*	1.26 (0.73–2.17)	1.00

*Note:* Low TIR was defined as TIR ≤ 70%, and high TIR was defined as TIR > 70%. Low 1,5‐AG was defined as 1,5‐AG < 6.0 μg/mL, and high 1,5‐AG was defined as 1,5‐AG ≥ 6.0 μg/mL. Model 1 included age and sex; Model 2 further included diabetes duration, body mass index, systolic blood pressure, triglycerides, high‐density lipoprotein cholesterol, low‐density lipoprotein cholesterol, current smoking status, estimated glomerular filtration rate, history of cancer, history of cardiovascular disease and the use of insulin, oral hypoglycemic drugs, antihypertensive drugs, lipid‐lowering drugs and antiplatelet drugs.

Abbreviations: 1,5‐AG, 1,5‐anhydroglucitol; CI, confidence interval; HR, hazard ratio; TIR, time in range (3.9–10.0 mmol/L).

* *p* < 0.05.

** *p* < 0.01.

*** *p* < 0.001.

### 3.3 Serum 1,5‐AG and All‐Cause Mortality Across TIR Subgroups

**FIGURE 2:**
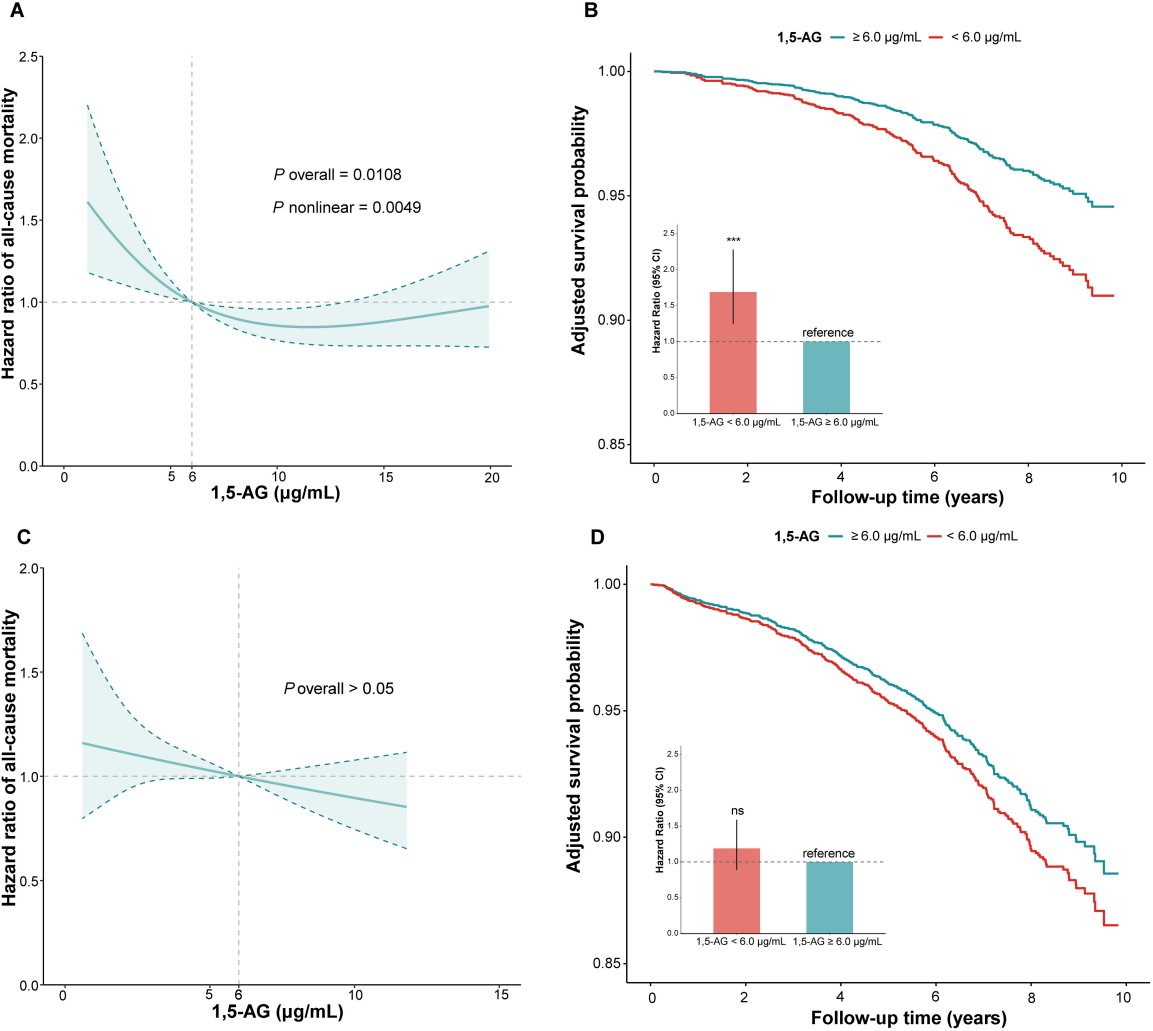
Serum 1,5‐AG and all‐cause mortality in participants stratified by TIR. Panel A: Dose–response relationship in participants with TIR > 70%. RCS analysis shows an L‐shaped association between 1,5‐AG and mortality, with risk significantly increasing below the threshold of approximately 6.0 μg/mL. Panel B: Adjusted survival curves in participants with TIR > 70%. Participants with 1,5‐AG < 6.0 μg/mL (red) had a significantly lower survival probability and higher mortality risk (HR 1.69, 95% CI 1.25–2.28) compared to those with ≥ 6.0 μg/mL (green). Panel C: Dose–response relationship in participants with TIR ≤ 70%. RCS analysis shows no significant association between 1,5‐AG levels and mortality risk. Panel D: Adjusted survival curves in participants with TIR ≤ 70%. There was no significant difference in survival probability (HR 1.19, 95% CI 0.89–1.59) between participants with 1,5‐AG < 6.0 μg/mL (red) and those with ≥ 6.0 μg/mL (green). Model included age, sex, diabetes duration, body mass index, systolic blood pressure, triglycerides, high‐density lipoprotein cholesterol, low‐density lipoprotein cholesterol, current smoking status, estimated glomerular filtration rate, history of cancer, history of cardiovascular disease, and the use of insulin, oral hypoglycemic drugs, antihypertensive drugs, lipid‐lowering drugs, and antiplatelet drugs. 1,5‐AG, 1,5‐anhydroglucitol; CI, confidence interval; HR, hazard ratio; TIR, time in range (3.9–10.0 mmol/L). ***p < 0.001.

**FIGURE 3:**
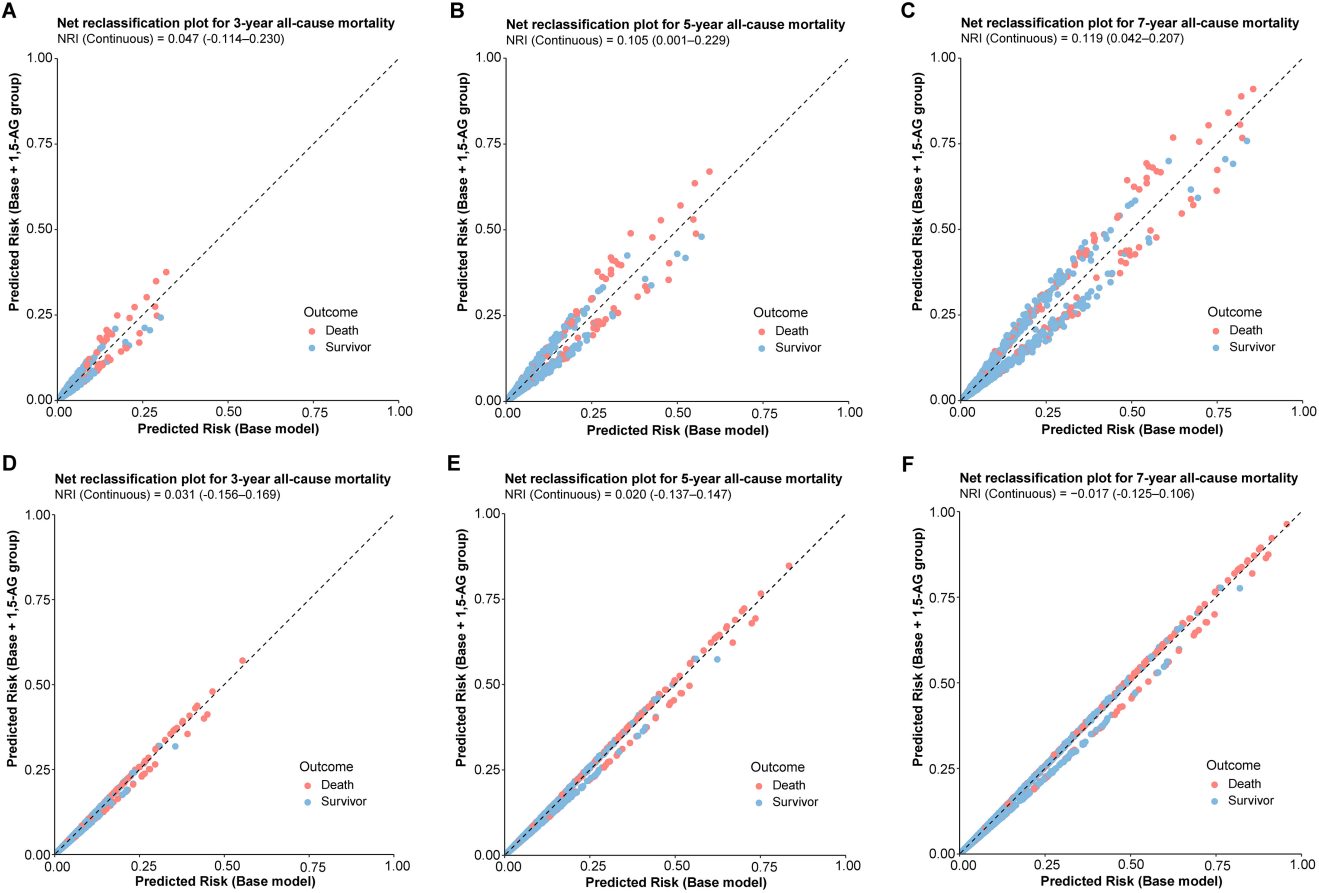
Net reclassification plots for all‐cause mortality across different time points in participants stratified by TIR. Scatter plots illustrate the reclassification of predicted mortality risk at 3, 5, and 7 years in participants with TIR > 70% (Panels A–C) and with TIR ≤ 70% (Panels D–F). The *x*‐axis represents the predicted risk from the base model, while the *y*‐axis represents the predicted risk from the model incorporating the 1,5‐AG group (threshold 6.0 μg/mL). Base model included age, sex, diabetes duration, body mass index, systolic blood pressure, triglycerides, high‐density lipoprotein cholesterol, low‐density lipoprotein cholesterol, current smoking status, estimated glomerular filtration rate, history of cancer, history of cardiovascular disease, and the use of insulin, oral hypoglycemic drugs, antihypertensive drugs, lipid‐lowering drugs, and antiplatelet drugs. 1,5‐AG, 1,5‐anhydroglucitol; NRI, net reclassification improvement; TIR, time in range (3.9–10.0 mmol/L).

Among participants achieving TIR > 70%, RCS analysis revealed an L‐shaped nonlinear association of serum 1,5‐AG with all‐cause mortality (*p* for nonlinearity = 0.0049), with risk increasing steeply below 6.0 μg/mL (Figure 2A). Consistent with this, participants with low 1,5‐AG (< 6.0 μg/mL) had significantly lower survival rates (Figure 2B). In fully adjusted Cox models (Model 2), low 1,5‐AG remained independently related to higher risk of all‐cause mortality (HR 1.69, 95% CI 1.25–2.28) (Figure 2B, Table S6). The results were generally consistent when serum 1,5‐AG was median‐categorized among participants with TIR > 70% (Table S7). There was no evidence of heterogeneity across prespecified subgroups stratified by age, sex, duration of diabetes, BMI, smoking status, history of cardiovascular disease and medication use (all *p* for interaction > 0.05, Table S8). Furthermore, the addition of 1,5‐AG to traditional risk factors (Model 2) improved reclassification for 5‐ and 7‐year mortality based on continuous NRIs (NRIs 0.105 and 0.119, respectively; both *p* < 0.05). The IDI was not significant at 5 years but reached statistical significance at 7‐year (IDI = 0.012, *p* < 0.05) (Figure 3A–C, Table S9).

In contrast, among patients with suboptimal glycemic control (TIR ≤ 70%), no apparent mortality gradient across serum 1,5‐AG levels was observed (Figure 2C,D). Cox regression indicated that low 1,5‐AG was not related to either all‐cause (HR 1.19, 95% CI 0.89–1.59) or cardiovascular mortality (HR 1.16, 95% CI 0.73–1.82) in this population (Table S6). Accordingly, adding 1,5‐AG to traditional risk factors yielded no improvement in reclassification for these patients (Figure 3D–F, Table S9).

### 3.4 Comparison of Serum 1,5‐AG and CGM‐Derived Glycemic Variability Indices

**FIGURE 4:**
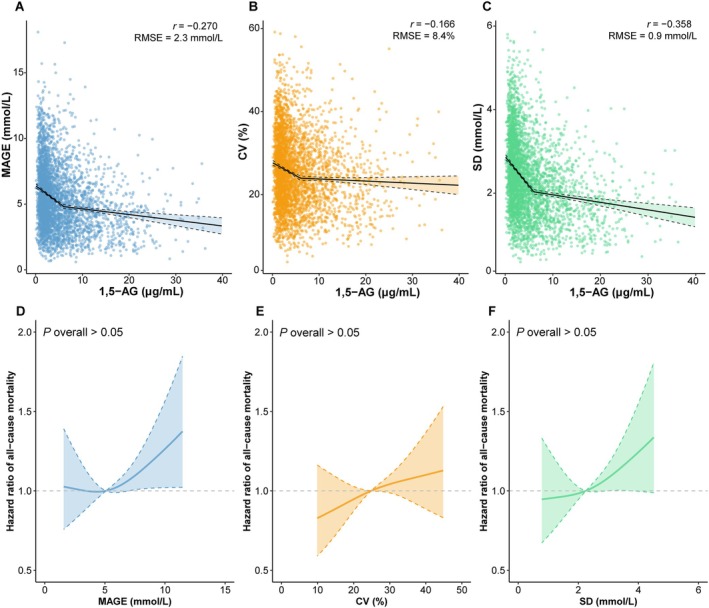
Serum 1,5‐AG and CGM‐derived glycemic variability metrics in the total population. Panels A–C: Associations between serum 1,5‐AG and CGM‐derived glycemic variability metrics including MAGE, CV, and SD, respectively. Solid lines represent fitted linear spline models with a knot at 6.0 μg/mL, and shaded areas indicate 95% confidence intervals. Spearman correlation coefficients (*r*) and root mean squared errors (RMSE) are shown for each association. Panels D–F: Restricted cubic spline analyses of MAGE, CV, and SD with all‐cause mortality. Solid lines indicate adjusted hazard ratios and shaded areas indicate 95% confidence intervals. Model included age, sex, diabetes duration, body mass index, systolic blood pressure, triglycerides, high‐density lipoprotein cholesterol, low‐density lipoprotein cholesterol, current smoking status, estimated glomerular filtration rate, history of cancer and history of cardiovascular disease, and the use of insulin, oral hypoglycemic drugs, antihypertensive drugs, lipid‐lowering drugs, and antiplatelet drugs. For the analysis of SD with all‐cause mortality, the model was additionally adjusted for mean sensor glucose. 1,5‐AG, 1,5‐anhydroglucitol; CGM, continuous glucose monitoring; CV, coefficient of variation; MAGE, mean amplitude of glycemic excursions; RMSE, root mean squared error; SD, standard deviation.

Serum 1,5‐AG was inversely correlated with CGM‐derived glycemic variability metrics, including MAGE (*r* = −0.270), CV (*r* = −0.166), and SD (*r* = −0.358) (all *p* < 0.05; Figure 4A–C). Scatter plots and fitted curves indicated that these metrics were elevated at lower 1,5‐AG levels and plateaued at higher levels. However, MAGE and CV were not associated with all‐cause mortality when modeled as continuous variables or tertiles, and SD was not significant after additional adjustment for MSG (Figure 4D–F, Table S10). Similar null associations were observed among participants with TIR > 70% (Figure S2).

## 4 Discussion

This large cohort study showed that the joint assessment of TIR and serum 1,5‐AG provided complementary prognostic information for both all‐cause and cardiovascular mortality. Among those who achieved TIR > 70%, reduced 1,5‐AG levels corresponded to lower survival probability, suggesting that 1,5‐AG may capture residual risk not fully captured by short‐term inpatient TIR.

As a clinically meaningful measure of glycemic control, TIR has shown consistent associations with the long‐term risk of diabetes‐related complications [29, 30, 31]. Recent data from 2752 US veterans also showed that reduced CGM‐derived TIR was related to elevated 5‐year risk of all‐cause death, independent of HbA1c [32]. Consistent with these findings, our analysis confirmed that TIR remained significantly related to the outcomes in the total cohort. However, TIR primarily quantifies the duration of time spent within the target range. Although it correlates strongly with HbA1c and mean glucose [8], TIR does not capture the magnitude or frequency of glucose excursions beyond target limits. Consequently, individuals with identical TIR values may exhibit markedly different degrees of glycemic variability [33]. In contrast, the measurement of serum 1,5‐AG may partly address this gap by reflecting the magnitude of glucose peaks that exceed the renal threshold of 10.0 mmol/L [19].

In the subgroup with TIR > 70%, we observed that serum 1,5‐AG remained related to all‐cause mortality after multivariable adjustment. This finding suggests that 1,5‐AG may provide complementary information not fully summarized by short‐term inpatient TIR, as it reflects recent hyperglycemic excursions over the preceding 1–2 weeks. Previous studies have reported consistent associations between 1,5‐AG and CGM‐derived glycemic measures, supporting its potential role as an adjunct to CGM and as a feasible marker of recent hyperglycemic excursions when CGM is unavailable [20, 34]. This explanation aligns with earlier evidence linking reduced 1,5‐AG levels to acute postprandial glucose excursions in individuals with prediabetes or well‐controlled type 2 diabetes [35]. These excursions may be clinically relevant by promoting oxidative stress and systemic inflammation [14].

Evidence from microvascular outcomes also supports the concept of residual risk. Kim et al. observed an approximately threefold higher prevalence of diabetic retinopathy among patients with HbA1c below 8.0% who had low 1,5‐AG levels [36]. In our previous work, reduced 1,5‐AG levels were also linked to a higher risk of diabetic retinopathy even among individuals who achieved adequate TIR [37]. The present study extends these observations from microvascular complications to long‐term mortality, suggesting that 1,5‐AG may identify residual risk beyond that reflected by conventional measures of glycemic control and CGM‐derived TIR. These findings may help identify patients who warrant closer monitoring and could be considered for interventional studies targeting glycemic excursions [19].

The incremental prediction analyses further indicated a potential complementary role of 1,5‐AG. In the TIR > 70% group, adding 1,5‐AG to the clinical model modestly improved continuous NRI at 5 and 7 years, whereas a significant improvement in IDI was observed only at 7 years. Given the statistical sensitivity of continuous NRI, these results should be interpreted cautiously. Even if its prognostic value is confirmed, the clinical use of 1,5‐AG may be limited by availability and regional cost differences. Future studies should validate these findings externally and assess the feasibility and cost‐effectiveness of its use in risk assessment.

By contrast, 1,5‐AG showed no significant relationship with mortality among participants with TIR ≤ 70%. However, because the interaction between TIR category and 1,5‐AG was not statistically significant, these subgroup findings should be regarded as exploratory rather than conclusive evidence of effect modification. It should also be acknowledged that tests for interaction generally require a larger number of events and may have insufficient statistical power, particularly in subgroup analyses. Nevertheless, a biologically plausible explanation may be considered. In participants with poor glycemic control, sustained glucose levels above the renal threshold may lead to continuous depletion of circulating 1,5‐AG [19]. As a result, serum 1,5‐AG concentrations may stabilize at very low levels and lose sensitivity to glycemic variability. Under these conditions, the marker shows significant overlap with indices of overall hyperglycemia such as TIR or HbA1c, and thus fails to contribute to the refinement of mortality risk.

In the joint classification analysis, participants with low TIR but high 1,5‐AG still showed a relatively high crude incidence rate of all‐cause mortality. This pattern may reflect sustained moderate hyperglycemia without frequent glucose peaks high enough to cause substantial glycosuria. As shown in Table 1, individuals with low TIR but high 1,5‐AG tended to be older and showed a longer diabetes duration, elevated systolic blood pressure, lower eGFR, and a higher prevalence of cardiovascular disease, indicating greater diabetes severity as well as cardiometabolic and renal vulnerability. This observation is also consistent with a previous CGM‐based stratification study showing that patients with hyperglycemia and low glycemic variability were characterized by pronounced insulin resistance [38]. However, given the relatively small size of this subgroup in our study, these findings should be interpreted cautiously and require further discussion. In this context, the mortality risk may be more closely related to cumulative glucotoxicity from sustained hyperglycemia, including oxidative stress and advanced glycation end‐product formation, which may promote endothelial dysfunction and macrovascular pathology [39]. Therefore, for patients with low TIR, increasing TIR to reduce the total hyperglycemic burden remains a priority.

Compared with 1,5‐AG, CGM metrics such as MAGE and CV showed no significant association with mortality. Although SD was initially associated with mortality, this association disappeared after additional adjustment for MSG, suggesting that it mainly reflects chronic hyperglycemia rather than glycemic variability. The different monitoring windows may partly explain these findings. CGM metrics typically reflect fluctuations during the recording window and are affected by short‐term changes in diet or lifestyle. In contrast, serum 1,5‐AG reflects cumulative exposure over 1–2 weeks, providing a more stable measure of glycemic variability. Additionally, 1,5‐AG allows repeated measurement during follow‐up, making it easier to track individual variability over time [40].

The present findings require consideration of several limitations. First, 1,5‐AG concentrations were assessed only at baseline, preventing longitudinal evaluation relative to disease progression. Second, the use of earlier‐generation CGM devices and a 3‐day monitoring period may have reduced the precision and reliability of glycemic variability estimates. The short assessment window may also have contributed to the observed discordance between TIR and 1,5‐AG. Although several CGM‐derived metrics can be estimated with acceptable reliability from relatively short recordings, longer monitoring periods provide a more representative assessment of usual glycemic patterns [41, 42]. In addition, participants were hospitalized patients from a single tertiary center and may have had greater clinical complexity than outpatient populations. Inpatient CGM metrics may also have been influenced by standardized meals, altered activity, and daily routines, limiting generalizability to community settings. Third, renal glucose handling may influence 1,5‐AG levels, and residual effects of renal dysfunction cannot be excluded despite adjustment for eGFR and consistent findings after excluding participants with eGFR < 30 mL/min/1.73 m^2^. Moreover, individuals receiving SGLT2 inhibitors were not included in the analysis. The applicability of our findings to patients receiving these agents therefore requires further evaluation, as SGLT2 inhibitors may reduce circulating 1,5‐AG concentrations through glycosuria. Nevertheless, evidence from the Atherosclerosis Risk in Communities cohort suggests that 1,5‐AG may help identify SGLT2 inhibitor use and adherence, indicating a potential use beyond glycemic monitoring [43]. Additional prospective data are warranted to clarify whether 1,5‐AG carries different prognostic and clinical implications in patients treated with SGLT2 inhibitors. Despite these constraints, the large sample size and detailed clinical data provide robust evidence for the clinical utility of TIR and 1,5‐AG.

Overall, both TIR and 1,5‐AG were prognostically relevant for long‐term mortality in type 2 diabetes. In participants with TIR > 70%, serum 1,5‐AG concentration below 6.0 μg/mL corresponded to greater all‐cause mortality risk, indicating that 1,5‐AG may complement TIR‐based assessment by revealing residual risk in this population.

## Author Contributions

F.J., T.X., and J.Z. conceived and designed the study, contributed to the interpretation of the data and revision of the manuscript, have full access to all the data in the study, and take responsibility for the integrity of the data and the accuracy of the data analysis. J.N. and H.S. performed statistical analysis and wrote the draft of the manuscript. L.C. cleaned the data and contributed to manuscript preparation. J.G. contributed to statistical analysis and reviewed the manuscript. C.W. and Y.W. contributed to data collection and reviewed the manuscript. J.L. reviewed and edited the manuscript. All authors read and approved the final manuscript.

## Funding

This work was funded by Noncommunicable Chronic Diseases‐National Science and Technology Major Project (2024ZD0532000, 2024ZD0532001), Shanghai Leading Talent Program of Eastern Talent Plan (LJ2024121), Shenzhen Medical Research Fund (C2406002), Sanming Project of Medicine in Shenzhen (SZSM202311019), Shanghai Action Plan for Science, Technology and Innovation (23JS1401000), and Shanghai Oriental Talent Program (Outstanding Project).

## Disclosure

The authors have nothing to report.

## Conflicts of Interest

The authors declare no conflicts of interest.

## Supporting information


**Figure S1:** Flowchart of the study.
**Figure S2:** Serum 1,5‐AG and CGM‐derived glycemic variability among participants with TIR > 70%.
**Table S1:** Associations of serum 1,5‐AG with all‐cause and cardiovascular mortality in total.
**Table S2:** Associations of TIR with all‐cause and cardiovascular mortality in total.
**Table S3:** Sensitivity analyses of the joint associations of TIR and serum 1,5‐AG with all‐cause and cardiovascular mortality after additional adjustment for glycemic metrics.
**Table S4:** Sensitivity analyses of the joint associations of TIR and serum 1,5‐AG with all‐cause and cardiovascular mortality after additional adjustment for baseline diabetic complications (*N* = 3236).
**Table S5:** Sensitivity analyses of the joint associations of TIR and serum 1,5‐AG with all‐cause and cardiovascular mortality after excluding participants with eGFR < 30 mL/min/1.73 m^2^ (*N* = 3652).
**Table S6:** Associations of serum 1,5‐AG with all‐cause and cardiovascular mortality among participants stratified by TIR.
**Table S7:** Sensitivity analyses of the associations of median‐based serum 1,5‐AG categories with all‐cause and cardiovascular mortality stratified by TIR.
**Table S8:** Subgroup analyses of the association between serum 1,5‐AG and all‐cause mortality among participants with TIR > 70%.
**Table S9:** Incremental predictive value of adding serum 1,5‐AG to Model 2 at different time points stratified by TIR.
**Table S10:** Associations of CGM‐derived glycemic variability metrics with all‐cause mortality in total.

## Data Availability

Restrictions apply to the availability of data generated or analyzed during this study to preserve patient confidentiality or because they were used under license. Data are, however, available from the authors upon reasonable request.

## References

[1] A. Ceriello and S. Colagiuri , “IDF Global Clinical Practice Recommendations for Managing Type 2 Diabetes – 2025,” Diabetes Research and Clinical Practice 222, no. S1 (2025): 112152, 10.1016/j.diabres.2025.112152. 40204549

[2] American Diabetes Association Professional Practice Committee for Diabetes, “6. Glycemic Goals, Hypoglycemia, and Hyperglycemic Crises: Standards of Care in Diabetes‐2026,” Diabetes Care 49, no. 1 (2026): S132–S149, 10.2337/dc26-S006. 41358894 PMC12690178

[3] American Diabetes Association Professional Practice Committee for Diabetes, “7.Diabetes Technology: Standards of Care in Diabetes‐2026,” Diabetes Care 49, no. 1 (2026): S150–S165, 10.2337/dc26-S007. 41358889 PMC12690173

[4] T. Battelino , C. M. Alexander , S. A. Amiel , et al., “Continuous Glucose Monitoring and Metrics for Clinical Trials: An International Consensus Statement,” Lancet Diabetes and Endocrinology 11, no. 1 (2023): 42–57, 10.1016/s2213-8587(22)00319-9. 36493795

[5] T. Battelino , T. Danne , R. M. Bergenstal , et al., “Clinical Targets for Continuous Glucose Monitoring Data Interpretation: Recommendations From the International Consensus on Time in Range,” Diabetes Care 42, no. 8 (2019): 1593–1603, 10.2337/dci19-0028. 31177185 PMC6973648

[6] J. Lu , X. Ma , Y. Shen , et al., “Time in Range Is Associated With Carotid Intima‐Media Thickness in Type 2 Diabetes,” Diabetes Technology & Therapeutics 22, no. 2 (2020): 72–78, 10.1089/dia.2019.0251. 31524497

[7] J. Lu , C. Wang , Y. Shen , et al., “Time in Range in Relation to All‐Cause and Cardiovascular Mortality in Patients With Type 2 Diabetes: A Prospective Cohort Study,” Diabetes Care 44, no. 2 (2021): 549–555, 10.2337/dc20-1862. 33097560 PMC9162101

[8] R. W. Beck , R. M. Bergenstal , P. Cheng , et al., “The Relationships Between Time in Range, Hyperglycemia Metrics, and HbA1c,” Journal of Diabetes Science and Technology 13, no. 4 (2019): 614–626, 10.1177/1932296818822496. 30636519 PMC6610606

[9] J. Lu , X. Ma , L. Zhang , et al., “Glycemic Variability Modifies the Relationship Between Time in Range and Hemoglobin A1c Estimated From Continuous Glucose Monitoring: A Preliminary Study,” Diabetes Research and Clinical Practice 161 (2020): 108032, 10.1016/j.diabres.2020.108032. 32006646

[10] A. Wang , X. Liu , J. Xu , et al., “Visit‐To‐Visit Variability of Fasting Plasma Glucose and the Risk of Cardiovascular Disease and All‐Cause Mortality in the General Population,” Journal of the American Heart Association 6, no. 12 (2017): e006757, 10.1161/jaha.117.006757. 29187392 PMC5779006

[11] M. Kuroda , T. Shinke , K. Sakaguchi , et al., “Association Between Daily Glucose Fluctuation and Coronary Plaque Properties in Patients Receiving Adequate Lipid‐Lowering Therapy Assessed by Continuous Glucose Monitoring and Optical Coherence Tomography,” Cardiovascular Diabetology 14 (2015): 78, 10.1186/s12933-015-0236-x. 26062762 PMC4480895

[12] M. Kuroda , T. Shinke , K. Sakaguchi , et al., “Effect of Daily Glucose Fluctuation on Coronary Plaque Vulnerability in Patients Pre‐Treated With Lipid‐Lowering Therapy: A Prospective Observational Study,” JACC. Cardiovascular Interventions 8, no. 6 (2015): 800–811, 10.1016/j.jcin.2014.11.025. 25999102

[13] F. Li , L. Zhang , Y. Shen , et al., “Higher Glucose Fluctuation Is Associated With a Higher Risk of Cardiovascular Disease: Insights From Pooled Results Among Patients With Diabetes,” Journal of Diabetes 15, no. 5 (2023): 368–381, 10.1111/1753-0407.13386. 37070713 PMC10172020

[14] L. Monnier , E. Mas , C. Ginet , et al., “Activation of Oxidative Stress by Acute Glucose Fluctuations Compared With Sustained Chronic Hyperglycemia in Patients With Type 2 Diabetes,” Journal of the American Medical Association 295, no. 14 (2006): 1681–1687, 10.1001/jama.295.14.1681. 16609090

[15] A. Ceriello , K. Esposito , L. Piconi , et al., “Oscillating Glucose Is More Deleterious to Endothelial Function and Oxidative Stress Than Mean Glucose in Normal and Type 2 Diabetic Patients,” Diabetes 57, no. 5 (2008): 1349–1354, 10.2337/db08-0063. 18299315

[16] L. Monnier , C. Colette , and D. Owens , “Glucose Variability and Diabetes Complications: Risk Factor or Biomarker? Can We Disentangle the “Gordian Knot”?,” Diabetes & Metabolism 47, no. 3 (May 2021): 101225, 10.1016/j.diabet.2021.101225. 33454438

[17] Y. Mo , J. Lu , and J. Zhou , “Glycemic Variability: Measurement, Target, Impact on Complications of Diabetes and Does It Really Matter?,” Journal of Diabetes Investigation 15, no. 1 (2024): 5–14, 10.1111/jdi.14112. 37988220 PMC10759720

[18] J. B. Buse , J. L. Freeman , S. V. Edelman , L. Jovanovic , and J. B. McGill , “Serum 1,5‐Anhydroglucitol (GlycoMark): A Short‐Term Glycemic Marker,” Diabetes Technology & Therapeutics 5, no. 3 (2003): 355–363, 10.1089/152091503765691839. 12828817

[19] K. M. Dungan , “1,5‐Anhydroglucitol (GlycoMark) as a Marker of Short‐Term Glycemic Control and Glycemic Excursions,” Expert Review of Molecular Diagnostics 8, no. 1 (2008): 9–19, 10.1586/14737159.8.1.9. 18088226

[20] H. Su , J. Ni , D. Peng , et al., “Association of Time in Tight Range and 1,5‐Anhydroglucitol in Type 2 Diabetes,” Diabetes, Obesity & Metabolism 27, no. 9 (2025): 4772–4781, 10.1111/dom.16515. PMC12326922 40490419

[21] K. M. Dungan , J. B. Buse , J. Largay , et al., “1,5‐Anhydroglucitol and Postprandial Hyperglycemia as Measured by Continuous Glucose Monitoring System in Moderately Controlled Patients With Diabetes,” Diabetes Care 29, no. 6 (2006): 1214–1219, 10.2337/dc06-1910. 16731998

[22] J. Ni , L. Chen , H. Su , et al., “Low Serum 1,5‐Anhydroglucitol Is Associated With Increased Risk of All‐Cause and Cardiovascular Mortality in Type 2 Diabetes: A Real‐World Cohort Study,” Diabetes, Obesity & Metabolism 28, no. 1 (2026): 434–442, 10.1111/dom.70214. PMC12673452 41090407

[23] J. Lu , X. Ma , J. Zhou , et al., “Association of Time in Range, as Assessed by Continuous Glucose Monitoring, With Diabetic Retinopathy in Type 2 Diabetes,” Diabetes Care 41, no. 11 (2018): 2370–2376, 10.2337/dc18-1131. 30201847

[24] A. S. Levey , L. A. Stevens , C. H. Schmid , et al., “A New Equation to Estimate Glomerular Filtration Rate,” Annals of Internal Medicine 150, no. 9 (2009): 604–612, 10.7326/0003-4819-150-9-200905050-00006. 19414839 PMC2763564

[25] J. Ni , W. Han , Y. Wang , et al., “The Relationship Between Glycated Albumin and Time in Tight Range in Type 2 Diabetes,” Journal of Diabetes 17, no. 3 (2025): e70073, 10.1111/1753-0407.70073. 40135648 PMC11938112

[26] E. Selvin , A. Rawlings , P. Lutsey , et al., “Association of 1,5‐Anhydroglucitol With Cardiovascular Disease and Mortality,” Diabetes 65, no. 1 (2016): 201–208, 10.2337/db15-0607. 26395741 PMC4686946

[27] J. Pan , X. Yan , F. Li , Y. Zhang , L. Jiang , and C. Wang , “Association of Glycemic Variability Assessed by Continuous Glucose Monitoring With Subclinical Diabetic Polyneuropathy in Type 2 Diabetes Patients,” Journal of Diabetes Investigation 13, no. 2 (2022): 328–335, 10.1111/jdi.13652. 34455710 PMC8847148

[28] H. Takahashi , N. Iwahashi , J. Kirigaya , et al., “Glycemic Variability Determined With a Continuous Glucose Monitoring System Can Predict Prognosis After Acute Coronary Syndrome,” Cardiovascular Diabetology 17, no. 1 (2018): 116, 10.1186/s12933-018-0761-5. 30121076 PMC6098663

[29] M. Yapanis , S. James , M. E. Craig , D. O'Neal , and E. I. Ekinci , “Complications of Diabetes and Metrics of Glycemic Management Derived From Continuous Glucose Monitoring,” Journal of Clinical Endocrinology and Metabolism 107, no. 6 (2022): e2221–e2236, 10.1210/clinem/dgac034. 35094087 PMC9113815

[30] J. De Meulemeester , S. Charleer , M. M. Visser , C. De Block , C. Mathieu , and P. Gillard , “The Association of Chronic Complications With Time in Tight Range and Time in Range in People With Type 1 Diabetes: A Retrospective Cross‐Sectional Real‐World Study,” Diabetologia 67, no. 8 (2024): 1527–1535, 10.1007/s00125-024-06171-y. 38787436

[31] L. Anagnostopoulou , A. L. Liarakos , I. Ntanasis‐Stathopoulos , A. Briasoulis , and A. Tentolouris , “Continuous Glucose Monitoring and Microvascular Complications in Diabetes: Bridging Glycemic Metrics With Clinical Outcomes,” Diabetes, Obesity & Metabolism 28, no. 2 (2026): 840–849, 10.1111/dom.70288. PMC12803653 41208627

[32] T. Okuno , S. A. Macwan , G. J. Norman , D. R. Miller , P. D. Reaven , and J. J. Zhou , “Continuous Glucose Monitoring Metrics Predict All‐Cause Mortality in Diabetes: A Real‐World Long‐Term Study,” Diabetes Care 48, no. 10 (2025): 1794–1802, 10.2337/dc25-0716. 40768053 PMC12451845

[33] Y. Wang , J. Lu , Y. Shen , et al., “Comparison of Glucose Time in Range and Area Under Curve in Range in Relation to Risk of Diabetic Retinopathy in Type 2 Diabetes Patients,” Journal of Diabetes Investigation 13, no. 9 (2022): 1543–1550, 10.1111/jdi.13811. 35435323 PMC9434583

[34] H. Su , J. Ni , J. Lu , et al., “Comparable Performance of 1,5‐Anhydroglucitol and Continuous Glucose Monitoring in Detecting Subclinical Atherosclerosis in Elderly Patients With Type 2 Diabetes,” Cardiovascular Diabetology 24, no. 1 (2025): 442, 10.1186/s12933-025-02993-1. 41272699 PMC12639912

[35] J. C. Won , C. Y. Park , H. S. Park , et al., “1,5‐Anhydroglucitol Reflects Postprandial Hyperglycemia and a Decreased Insulinogenic Index, Even in Subjects With Prediabetes and Well‐Controlled Type 2 Diabetes,” Diabetes Research and Clinical Practice 84, no. 1 (2009): 51–57, 10.1016/j.diabres.2009.01.002. 19187997

[36] W. J. Kim , C. Y. Park , S. E. Park , et al., “Serum 1,5‐Anhydroglucitol Is Associated With Diabetic Retinopathy in Type 2 Diabetes,” Diabetic Medicine 29, no. 9 (2012): 1184–1190, 10.1111/j.1464-5491.2012.03613.x. 22332964

[37] H. Su , J. Ni , J. Lu , et al., “1,5‐Anhydroglucitol and Time in Range as Complementary Markers for Diabetic Retinopathy in Type 2 Diabetes,” Diabetes/Metabolism Research and Reviews 42, no. 3 (2026): e70152, 10.1002/dmrr.70152. 41791794 PMC12965829

[38] X. Shao , J. Lu , R. Tao , et al., “Clinically Relevant Stratification of Patients With Type 2 Diabetes by Using Continuous Glucose Monitoring Data,” Diabetes, Obesity & Metabolism 26, no. 6 (2024): 2082–2091, 10.1111/dom.15512. 38409633

[39] X. Luo , J. Wu , S. Jing , and L. J. Yan , “Hyperglycemic Stress and Carbon Stress in Diabetic Glucotoxicity,” Aging and Disease 7, no. 1 (2016): 90–110, 10.14336/ad.2015.0702. 26816666 PMC4723237

[40] L. Liu , X. Wan , J. Liu , Z. Huang , X. Cao , and Y. Li , “Increased 1,5‐Anhydroglucitol Predicts Glycemic Remission in Patients With Newly Diagnosed Type 2 Diabetes Treated With Short‐Term Intensive Insulin Therapy,” Diabetes Technology & Therapeutics 14, no. 9 (2012): 756–761, 10.1089/dia.2012.0055. 22731793 PMC3429328

[41] Y. D. Foreman , M. Brouwers , C. J. H. van der Kallen , et al., “Glucose Variability Assessed With Continuous Glucose Monitoring: Reliability, Reference Values, and Correlations With Established Glycemic Indices‐The Maastricht Study,” Diabetes Technology & Therapeutics 22, no. 5 (2020): 395–403, 10.1089/dia.2019.0385. 31886732

[42] T. D. Riddlesworth , R. W. Beck , R. L. Gal , et al., “Optimal Sampling Duration for Continuous Glucose Monitoring to Determine Long‐Term Glycemic Control,” Diabetes Technology & Therapeutics 20, no. 4 (2018): 314–316, 10.1089/dia.2017.0455. 29565197

[43] N. R. Daya , M. Fang , M. Mathew , et al., “1,5‐Anhydroglucitol: A Novel Biomarker of Adherence to Sodium‐Glucose Cotransporter 2 Inhibitors,” Diabetes Care 47, no. 2 (2024): e9–e10, 10.2337/dc23-2035. 38091486 PMC10834388

