# Supplementary material for: Serum 1,5‐Anhydroglucitol Identifies Residual Mortality Risk Beyond Time in Range in Type 2 Diabetes: A Cohort Study
Source: J Diabetes. 2026 Aug 4;18(8):e70260. doi: 10.1111/1753-0407.70260 (PMC13437425; doi:10.1111/1753-0407.70260)
Supplement: Supplementary file 1 [file JDB-18-e70260-s001.docx]

**Supplementary materials**

[**Supplementary Figure 1.** Flowchart of the study	2](#_Toc235277898)

[**Supplementary Figure 2.** Serum 1,5-AG and CGM-derived glycemic variability among participants with TIR >70%	3](#_Toc235277899)

[**Supplementary Table 1.** Associations of serum 1,5-AG with all-cause and cardiovascular mortality in total	4](#_Toc235277900)

[**Supplementary Table 2.** Associations of TIR with all-cause and cardiovascular mortality in total	5](#_Toc235277901)

[**Supplementary Table 3.** Sensitivity analyses of the joint associations of TIR and serum 1,5-AG with all-cause and cardiovascular mortality after additional adjustment for glycemic metrics	6](#_Toc235277902)

[**Supplementary Table 4.** Sensitivity analyses of the joint associations of TIR and serum 1,5-AG with all-cause and cardiovascular mortality after additional adjustment for baseline diabetic complications (N = 3,236)	8](#_Toc235277903)

[**Supplementary Table 5.** Sensitivity analyses of the joint associations of TIR and serum 1,5-AG with all-cause and cardiovascular mortality after excluding participants with eGFR <30 mL/min/1.73 m² (N = 3,652)	10](#_Toc235277904)

[**Supplementary Table 6.** Associations of serum 1,5-AG with all-cause and cardiovascular mortality among participants stratified by TIR	12](#_Toc235277905)

[**Supplementary Table 7.** Sensitivity analyses of the associations of median-based serum 1,5-AG categories with all-cause and cardiovascular mortality stratified by TIR	14](#_Toc235277906)

[**Supplementary Table 8.** Subgroup analyses of the association between serum 1,5-AG and all-cause mortality among participants with TIR >70%	16](#_Toc235277907)

[**Supplementary Table 9**. Incremental predictive value of adding serum 1,5-AG to Model 2 at different time points stratified by TIR	17](#_Toc235277908)

[**Supplementary Table 10.** Associations of CGM-derived glycemic variability metrics with all-cause mortality in total	18](#_Toc235277909)

# **Supplementary Figure 1. Flowchart of the study**

Abbreviations: CGM, continuous glucose monitoring; SGLT2, sodium-glucose cotransporter-2.

# **Supplementary Figure 2. Serum 1,5-AG and CGM-derived glycemic variability among participants with TIR >70%**

Panels A-C: Associations between serum 1,5-AG and MAGE, CV or SD among participants with TIR > 70%. Solid lines represent fitted linear spline models with a knot at 6.0 μg/mL, and shaded areas indicate 95% confidence intervals. Spearman correlation coefficients (*r*) and root mean squared errors (RMSE) are shown for each association.

Panels D-F: Restricted cubic spline analyses of MAGE, CV, and SD with all-cause mortality among participants with TIR > 70%. Solid lines indicate adjusted hazard ratios and shaded areas indicate 95% confidence intervals.

*Notes:* Model adjusted for age, sex, diabetes duration, body mass index, systolic blood pressure, triglycerides, high-density lipoprotein cholesterol, low-density lipoprotein cholesterol, current smoking status, estimated glomerular filtration rate, history of cancer, history of cardiovascular disease, and the use of insulin, oral hypoglycemic drugs, antihypertensive drugs, lipid-lowering drugs and antiplatelet drugs. For the analysis of SD with all-cause mortality, the model was additionally adjusted for mean sensor glucose.

Abbreviations: 1,5-AG, 1,5-anhydroglucitol; CGM, continuous glucose monitoring; CV, coefficient of variation; MAGE, mean amplitude of glycemic excursions; RMSE, root mean squared errors; SD, standard deviation; TIR, time in range (3.9–10.0 mmol/L).

# **Supplementary Table 1. Associations of serum 1,5-AG with all-cause and cardiovascular mortality in total**

|  | 1,5-AG (μg/mL) | | 1,5-AG as a continuous variable  (each 1 SD decrease) |
| --- | --- | --- | --- |
|  | ≥ 6.0 | < 6.0 |  |
| **All-cause mortality** |  |  |  |
| Number of participants | 1083 | 2594 |  |
| Number of deaths | 133 | 389 |  |
| Person-years | 7986.72 | 18417.97 |  |
| Incidence rate (per 1000 person-years) | 16.65 | 21.12 |  |
| HRs (95% CIs) |  |  |  |
| Unadjusted | 1.00 | 1.29 (1.06-1.57) ^*^ | 1.07 (0.98-1.18) |
| Model 1 | 1.00 | 1.45 (1.19-1.77) ^***^ | 1.16 (1.05-1.28) ^**^ |
| Model 2 | 1.00 | 1.49 (1.22-1.82) ^***^ | 1.17 (1.06-1.30) ^**^ |
| **Cardiovascular mortality** |  |  |  |
| Number of deaths | 48 | 133 |  |
| Incidence rate (per 1000 person-years) | 6.01 | 7.22 |  |
| HRs (95% CIs) |  |  |  |
| Unadjusted | 1.00 | 1.23 (0.88-1.70) | 1.14 (0.96-1.34) |
| Model 1 | 1.00 | 1.41 (1.01-1.96) ^*^ | 1.26 (1.05-1.51) ^*^ |
| Model 2 | 1.00 | 1.42 (1.02-1.99) ^*^ | 1.28 (1.06-1.55) ^**^ |

*Notes:* Model 1 included age and sex; Model 2 further included diabetes duration, body mass index, systolic blood pressure, triglycerides, high-density lipoprotein cholesterol, low-density lipoprotein cholesterol, current smoking status, estimated glomerular filtration rate, history of cancer, history of cardiovascular disease, and the use of insulin, oral hypoglycemic drugs, antihypertensive drugs, lipid-lowering drugs and antiplatelet drugs.

Abbreviations: 1,5-AG, 1,5-anhydroglucitol; CI, confidence interval; HR, hazard ratio; SD, standard deviation. ^*^*P* < 0.05, ^**^*P* < 0.01, ^***^*P* < 0.001

# **Supplementary Table 2. Associations of TIR with all-cause and cardiovascular mortality in total**

|  | TIR (%) | | TIR as a continuous variable  (each 1 SD decrease) |
| --- | --- | --- | --- |
|  | > 70 | ≤ 70 |  |
| **All-cause mortality** |  |  |  |
| Number of participants | 1699 | 1978 |  |
| Number of deaths | 190 | 332 |  |
| Person-years | 12467.28 | 13937.40 |  |
| Incidence rate (per 1000 person-years) | 15.24 | 23.82 |  |
| HRs (95% CIs) |  |  |  |
| Unadjusted | 1.00 | 1.58 (1.32-1.89) ^***^ | 1.24 (1.14-1.34) ^***^ |
| Model 1 | 1.00 | 1.37 (1.15-1.64) ^***^ | 1.14 (1.05-1.24) ^**^ |
| Model 2 | 1.00 | 1.33 (1.10-1.60) ^**^ | 1.12 (1.02-1.22) ^*^ |
| **Cardiovascular mortality** |  |  |  |
| Number of deaths | 53 | 128 |  |
| Incidence rate (per 1000 person-years) | 4.25 | 9.18 |  |
| HRs (95% CIs) |  |  |  |
| Unadjusted | 1.00 | 2.19 (1.59-3.02) ^***^ | 1.40 (1.22-1.60) ^***^ |
| Model 1 | 1.00 | 1.86 (1.34-2.56) ^***^ | 1.26 (1.10-1.45) ^***^ |
| Model 2 | 1.00 | 1.80 (1.29-2.51) ^***^ | 1.25 (1.08-1.45) ^**^ |

*Notes:* Model 1 included age and sex; Model 2 further included diabetes duration, body mass index, systolic blood pressure, triglycerides, high-density lipoprotein cholesterol, low-density lipoprotein cholesterol, current smoking status, estimated glomerular filtration rate, history of cancer, history of cardiovascular disease, and the use of insulin, oral hypoglycemic drugs, antihypertensive drugs, lipid-lowering drugs and antiplatelet drugs.

Abbreviations: CI, confidence interval; HR, hazard ratio; SD, standard deviation; TIR, time in range (3.9–10.0 mmol/L). ^*^*P* < 0.05, ^**^*P* < 0.01, ^***^*P* < 0.001

# **Supplementary Table 3. Sensitivity analyses of the joint associations of TIR and serum 1,5-AG with all-cause and cardiovascular mortality after additional adjustment for glycemic metrics**

|  | Group | | | |
| --- | --- | --- | --- | --- |
|  | low TIR/low 1,5-AG | low TIR/high 1,5-AG | high TIR/low 1,5-AG | high TIR/high 1,5-AG |
| **All-cause mortality** |  |  |  |  |
| Number of participants | 1695 | 283 | 899 | 800 |
| Number of deaths | 275 | 57 | 114 | 76 |
| Person-years | 11944.03 | 1993.37 | 6473.94 | 5993.34 |
| Incidence rate (per 1000 person-years) | 23.02 | 28.59 | 17.61 | 12.68 |
| HRs (95% CIs) |  |  |  |  |
| Model 2 | 1.82 (1.40-2.37) ^***^ | 1.58 (1.11-2.24) ^*^ | 1.69 (1.26-2.27) ^***^ | 1.00 |
| Model 3 | 1.38 (1.03-1.85) ^*^ | 1.46 (1.03-2.08) ^*^ | 1.40 (1.03-1.91) ^*^ | 1.00 |
| Model 4 | 1.72 (1.31-2.26) ^***^ | 1.53 (1.08-2.18) ^*^ | 1.66 (1.24-2.23) ^***^ | 1.00 |
| Model 5 | 1.85 (1.29-2.64) ^***^ | 1.60 (1.08-2.36) ^*^ | 1.69 (1.26-2.27) ^***^ | 1.00 |
| Model 6 | 1.82 (1.40-2.36) ^***^ | 1.59 (1.12-2.25) ^**^ | 1.69 (1.26-2.26) ^***^ | 1.00 |
| **Cardiovascular mortality** |  |  |  |  |
| Number of deaths | 105 | 23 | 28 | 25 |
| Incidence rate (per 1000 person-years) | 8.79 | 11.54 | 4.33 | 4.17 |
| HRs (95% CIs) |  |  |  |  |
| Model 2 | 2.09 (1.33-3.27) ^**^ | 1.80 (1.01-3.21) ^*^ | 1.26 (0.73-2.17) | 1.00 |
| Model 3 | 1.46 (0.89-2.41) | 1.63 (0.91-2.90) | 1.00 (0.57-1.76) | 1.00 |
| Model 4 | 1.98 (1.24-3.16) ^**^ | 1.76 (0.98-3.14) | 1.25 (0.72-2.15) | 1.00 |
| Model 5 | 1.85 (1.01-3.37) ^*^ | 1.65 (0.86-3.15) | 1.25 (0.73-2.15) | 1.00 |
| Model 6 | 2.01 (1.28-3.15) ^**^ | 1.79 (1.00-3.18) ^*^ | 1.23 (0.71-2.12) | 1.00 |

*Notes:* Low TIR was defined as TIR ≤70%, and high TIR was defined as TIR >70%. Low 1,5-AG was defined as 1,5-AG <6.0 μg/mL, and high 1,5-AG was defined as 1,5-AG ≥6.0 μg/mL. Model 2 was adjusted for age, sex, diabetes duration, body mass index, systolic blood pressure, triglycerides, high-density lipoprotein cholesterol, low-density lipoprotein cholesterol, current smoking status, estimated glomerular filtration rate, history of cancer, history of cardiovascular disease, and the use of insulin, oral hypoglycemic drugs, antihypertensive drugs, lipid-lowering drugs, and antiplatelet drugs. Additional sensitivity models were further adjusted for HbA1c (Model 3), fasting plasma glucose (Model 4), time above range (Model 5), and time below range (Model 6), respectively.

Abbreviations: 1,5-AG, 1,5-anhydroglucitol; CI, confidence interval; HbA1c, glycated hemoglobin A1c; HR, hazard ratio; TIR, time in range (3.9–10.0 mmol/L). ^*^*P* < 0.05, ^**^*P* < 0.01, ^***^*P* < 0.001

# **Supplementary Table 4. Sensitivity analyses of the joint associations of TIR and serum 1,5-AG with all-cause and cardiovascular mortality after additional adjustment for baseline diabetic complications (N = 3,236)**

|  | Group | | | |
| --- | --- | --- | --- | --- |
|  | low TIR/low 1,5-AG | low TIR/high 1,5-AG | high TIR/low 1,5-AG | high TIR/high 1,5-AG |
| **All-cause mortality** |  |  |  |  |
| Number of participants | 1492 | 241 | 792 | 711 |
| Number of deaths | 221 | 45 | 92 | 63 |
| Person-years | 10557.59 | 1697.76 | 5718.90 | 5371.58 |
| Incidence rate (per 1000 person-years) | 20.93 | 26.51 | 16.09 | 11.73 |
| HRs (95% CIs) |  |  |  |  |
| Unadjusted | 1.83 (1.38-2.42) ^***^ | 2.32 (1.58-3.40) ^***^ | 1.40 (1.02-1.93) ^*^ | 1.00 |
| Model 1 | 1.81 (1.36-2.39) ^***^ | 1.69 (1.16-2.49) ^**^ | 1.67 (1.21-2.31) ^**^ | 1.00 |
| Model 2 | 1.80 (1.34-2.40) ^***^ | 1.56 (1.06-2.30) ^*^ | 1.66 (1.20-2.30) ^**^ | 1.00 |
| Model 3 | 1.69 (1.27-2.26) ^***^ | 1.50 (1.02-2.22) ^*^ | 1.53 (1.11-2.12) ^**^ | 1.00 |
| **Cardiovascular mortality** |  |  |  |  |
| Number of deaths | 86 | 19 | 21 | 23 |
| Incidence rate (per 1000 person-years) | 8.15 | 11.19 | 3.67 | 4.28 |
| HRs (95% CIs) |  |  |  |  |
| Unadjusted | 1.96 (1.24-3.10) ^**^ | 2.70 (1.47-4.96) ^**^ | 0.88 (0.49-1.59) | 1.00 |
| Model 1 | 1.95 (1.23-3.09) ^**^ | 1.89 (1.03-3.47) ^*^ | 1.10 (0.61-1.99) | 1.00 |
| Model 2 | 1.79 (1.11-2.87) ^*^ | 1.63 (0.88-3.04) | 1.02 (0.56-1.85) | 1.00 |
| Model 3 | 1.69 (1.05-2.72) ^*^ | 1.61 (0.87-3.00) | 0.94 (0.52-1.71) | 1.00 |

*Notes:* This analysis was conducted among participants with complete data on urinary albumin and fundus examination. Albuminuria was defined as urine albumin-creatinine ratio >30 mg/g or 24-hour urine albumin excretion >30 mg/24h. Low TIR was defined as TIR ≤70%, and high TIR was defined as TIR >70%. Low 1,5-AG was defined as 1,5-AG <6.0 μg/mL, and high 1,5-AG was defined as 1,5-AG ≥6.0 μg/mL. Model 1 included age and sex; Model 2 further included diabetes duration, body mass index, systolic blood pressure, triglycerides, high-density lipoprotein cholesterol, low-density lipoprotein cholesterol, current smoking status, estimated glomerular filtration rate, history of cancer, history of cardiovascular disease, and the use of insulin, oral hypoglycemic drugs, antihypertensive drugs, lipid-lowering drugs and antiplatelet drugs; Model 3 was further adjusted for baseline albuminuria and diabetic retinopathy.

Abbreviations: 1,5-AG, 1,5-anhydroglucitol; CI, confidence interval; HR, hazard ratio; TIR, time in range (3.9–10.0 mmol/L). ^*^*P* < 0.05, ^**^*P* < 0.01, ^***^*P* < 0.001

# **Supplementary Table 5. Sensitivity analyses of the joint associations of TIR and serum 1,5-AG with all-cause and cardiovascular mortality after excluding participants with eGFR <30 mL/min/1.73 m² (N = 3,652)**

|  | Group | | | |
| --- | --- | --- | --- | --- |
|  | low TIR/low 1,5-AG | low TIR/high 1,5-AG | high TIR/low 1,5-AG | high TIR/high 1,5-AG |
| **All-cause mortality** |  |  |  |  |
| Number of participants | 1683 | 281 | 893 | 795 |
| Number of deaths | 266 | 55 | 109 | 72 |
| Person-years | 11879.99 | 1984.99 | 6445.14 | 5962.04 |
| Incidence rate (per 1000 person-years) | 22.39 | 27.71 | 16.91 | 12.08 |
| HRs (95% CIs) |  |  |  |  |
| Unadjusted | 1.90 (1.46-2.46) ^***^ | 2.35 (1.65-3.34) ^***^ | 1.43 (1.06-1.93) ^*^ | 1.00 |
| Model 1 | 1.85 (1.43-2.41) ^***^ | 1.74 (1.22-2.47) ^**^ | 1.66 (1.24-2.24) ^***^ | 1.00 |
| Model 2 | 1.84 (1.41-2.41) ^***^ | 1.58 (1.11-2.26) ^*^ | 1.69 (1.25-2.29) ^***^ | 1.00 |
| **Cardiovascular mortality** |  |  |  |  |
| Number of deaths | 102 | 21 | 25 | 25 |
| Incidence rate (per 1000 person-years) | 8.59 | 10.58 | 3.88 | 4.19 |
| HRs (95% CIs) |  |  |  |  |
| Unadjusted | 2.11 (1.36-3.27) ^***^ | 2.60 (1.46-4.65) ^**^ | 0.95 (0.55-1.66) | 1.00 |
| Model 1 | 2.07 (1.33-3.21) ^**^ | 1.83 (1.02-3.27) ^*^ | 1.15 (0.66-2.00) | 1.00 |
| Model 2 | 2.07 (1.32-3.25) ^**^ | 1.60 (0.89-2.89) | 1.15 (0.66-2.01) | 1.00 |

*Notes:* Low TIR was defined as TIR ≤70%, and high TIR was defined as TIR >70%. Low 1,5-AG was defined as 1,5-AG <6.0 μg/mL, and high 1,5-AG was defined as 1,5-AG ≥6.0 μg/mL. Model 1 was adjusted for age and sex. Model 2 was further adjusted for diabetes duration, body mass index, systolic blood pressure, triglycerides, high-density lipoprotein cholesterol, low-density lipoprotein cholesterol, current smoking status, estimated glomerular filtration rate, history of cancer, history of cardiovascular disease, and the use of insulin, oral hypoglycemic drugs, antihypertensive drugs, lipid-lowering drugs, and antiplatelet drugs.

Abbreviations: 1,5-AG, 1,5-anhydroglucitol; CI, confidence interval; eGFR, estimated glomerular filtration rate; HR, hazard ratio; TIR, time in range (3.9–10.0 mmol/L). ^*^*P* < 0.05, ^**^*P* < 0.01, ^***^*P* < 0.001

# **Supplementary Table 6. Associations of serum 1,5-AG with all-cause and cardiovascular mortality among participants stratified by TIR**

|  | 1,5-AG (μg/mL) | |
| --- | --- | --- |
|  | ≥ 6.0 | < 6.0 |
| **TIR > 70%** |  |  |
| **All-cause mortality** |  |  |
| Number of participants | 800 | 899 |
| Number of deaths | 76 | 114 |
| Person-years | 5993.34 | 6473.94 |
| Incidence rate (per 1000 person-years) | 12.68 | 17.61 |
| HRs (95% CIs) |  |  |
| Unadjusted | 1.00 | 1.43 (1.07-1.91) ^*^ |
| Model 1 | 1.00 | 1.65 (1.23-2.21) ^***^ |
| Model 2 | 1.00 | 1.69 (1.25-2.28) ^***^ |
| **Cardiovascular mortality** |  |  |
| Number of deaths | 25 | 28 |
| Incidence rate (per 1000 person-years) | 4.17 | 4.33 |
| HRs (95% CIs) |  |  |
| Unadjusted | 1.00 | 1.07 (0.62-1.83) |
| Model 1 | 1.00 | 1.31 (0.76-2.26) |
| Model 2 | 1.00 | 1.22 (0.70-2.14) |
|  |  |  |
| **TIR ≤ 70%** |  |  |
| **All-cause mortality** |  |  |
| Number of participants | 283 | 1695 |
| Number of deaths | 57 | 275 |
| Person-years | 1993.37 | 11944.03 |
| Incidence rate (per 1000 person-years) | 28.59 | 23.02 |
| HRs (95% CIs) |  |  |
| Unadjusted | 1.00 | 0.80 (0.60-1.07) |
| Model 1 | 1.00 | 1.07 (0.80-1.42) |
| Model 2 | 1.00 | 1.19 (0.89-1.59) |
| **Cardiovascular mortality** |  |  |
| Number of deaths | 23 | 105 |
| Incidence rate (per 1000 person-years) | 11.54 | 8.79 |
| HRs (95% CIs) |  |  |
| Unadjusted | 1.00 | 0.76 (0.48-1.20) |
| Model 1 | 1.00 | 1.06 (0.67-1.66) |
| Model 2 | 1.00 | 1.16 (0.73-1.82) |

*Notes:* Model 1 included age and sex; Model 2 further included diabetes duration, body mass index, systolic blood pressure, triglycerides, high-density lipoprotein cholesterol, low-density lipoprotein cholesterol, current smoking status, estimated glomerular filtration rate, history of cancer and history of cardiovascular disease, and the use of insulin, oral hypoglycemic drugs, antihypertensive drugs, lipid-lowering drugs and antiplatelet drugs.

Abbreviations: 1,5-AG, 1,5-anhydroglucitol; CI, confidence interval; HR, hazard ratio; TIR, time in range (3.9–10.0 mmol/L). ^*^*P* < 0.05, ^**^*P* < 0.01, ^***^*P* < 0.001

# **Supplementary Table 7. Sensitivity analyses of the associations of median-based serum 1,5-AG categories with all-cause and cardiovascular mortality stratified by TIR**

|  | 1,5-AG (μg/mL) | |
| --- | --- | --- |
|  | ≥3.3 | <3.3 |
| **TIR > 70%** |  |  |
| **All-cause mortality** |  |  |
| Number of participants | 1185 | 514 |
| Number of deaths | 126 | 64 |
| Person-years | 8781.99 | 3685.29 |
| Incidence rate (per 1000 person-years) | 14.35 | 17.37 |
| HRs (95% CIs) |  |  |
| Unadjusted | 1.00 | 1.24 (0.92-1.68) |
| Model 1 | 1.00 | 1.61 (1.19-2.18) ^**^ |
| Model 2 | 1.00 | 1.59 (1.16-2.18) ^**^ |
| **Cardiovascular mortality** |  |  |
| Number of deaths | 34 | 19 |
| Incidence rate (per 1000 person-years) | 3.87 | 5.16 |
| HRs (95% CIs) |  |  |
| Unadjusted | 1.00 | 1.37 (0.78-2.40) |
| Model 1 | 1.00 | 1.95 (1.11-3.43) ^*^ |
| Model 2 | 1.00 | 1.88 (1.04-3.41) ^*^ |
|  |  |  |
| **TIR ≤ 70%** |  |  |
| **All-cause mortality** |  |  |
| Number of participants | 659 | 1319 |
| Number of deaths | 130 | 202 |
| Person-years | 4686.61 | 9250.79 |
| Incidence rate (per 1000 person-years) | 27.74 | 21.84 |
| HRs (95% CIs) |  |  |
| Unadjusted | 1.00 | 0.79 (0.63-0.99) ^*^ |
| Model 1 | 1.00 | 1.09 (0.87-1.36) |
| Model 2 | 1.00 | 1.16 (0.93-1.46) |
| **Cardiovascular mortality** |  |  |
| Number of deaths | 48 | 80 |
| Incidence rate (per 1000 person-years) | 10.24 | 8.65 |
| HRs (95% CIs) |  |  |
| Unadjusted | 1.00 | 0.85 (0.59-1.22) |
| Model 1 | 1.00 | 1.23 (0.85-1.76) |
| Model 2 | 1.00 | 1.36 (0.94-1.96) |

*Notes:* Model 1 included age and sex; Model 2 further included diabetes duration, body mass index, systolic blood pressure, triglycerides, high-density lipoprotein cholesterol, low-density lipoprotein cholesterol, current smoking status, estimated glomerular filtration rate, history of cancer, history of cardiovascular disease, and the use of insulin, oral hypoglycemic drugs, antihypertensive drugs, lipid-lowering drugs and antiplatelet drugs.

Abbreviations: 1,5-AG, 1,5-anhydroglucitol; CI, confidence interval; HR, hazard ratio; TIR, time in range (3.9–10.0 mmol/L). ^*^*P* < 0.05, ^**^*P* < 0.01, ^***^*P* < 0.001

# **Supplementary Table 8. Subgroup analyses of the association between serum 1,5-AG and all-cause mortality among participants with TIR >70%**

| Subgroup | Participants/  deaths, n | HR (95%CI) | *P* for interaction |
| --- | --- | --- | --- |
| Sex |  |  | 0.386 |
| Male | 948/131 | 1.94 (1.33-2.83) ^***^ |  |
| Female | 751/59 | 1.29 (0.75-2.21) |  |
| Age |  |  | 0.558 |
| ≥ 60 years | 973/157 | 1.68 (1.21-2.32) ^**^ |  |
| < 60 years | 726/33 | 1.13 (0.51-2.52) |  |
| Diabetes duration |  |  | 0.162 |
| ≥ 10 years | 919/133 | 1.53 (1.07-2.19) ^*^ |  |
| < 10 years | 780/57 | 2.06 (1.16-3.66) ^*^ |  |
| Body mass index |  |  | 0.879 |
| ≥ 24 kg/m^2^ | 1072/113 | 1.73 (1.16-2.57) ^**^ |  |
| < 24 kg/m^2^ | 627/77 | 1.77 (1.10-2.86) ^*^ |  |
| Current smoker |  |  | 0.474 |
| Yes | 421/64 | 1.91 (1.07-3.42) ^*^ |  |
| No | 1278/126 | 1.52 (1.05-2.19) ^*^ |  |
| History of cardiovascular disease |  |  | 0.939 |
| Yes | 396/84 | 1.84 (1.16-2.92) ^**^ |  |
| No | 1303/106 | 1.61 (1.07-2.42) ^*^ |  |
| Insulin |  |  | 0.902 |
| Use | 663/109 | 1.72 (1.15-2.60) ^**^ |  |
| No use | 1036/81 | 1.61 (1.01-2.55) ^*^ |  |
| Antihypertensive drugs |  |  | 0.625 |
| Use | 952/138 | 1.78 (1.25-2.53) ^**^ |  |
| No use | 747/52 | 1.38 (0.76-2.49) |  |

*Notes:* Model included age, sex, diabetes duration, body mass index, systolic blood pressure, triglycerides, high-density lipoprotein cholesterol, low-density lipoprotein cholesterol, current smoking status, estimated glomerular filtration rate, history of cancer, history of cardiovascular disease, and the use of insulin, oral hypoglycemic drugs, antihypertensive drugs, lipid-lowering drugs and antiplatelet drugs. The reference group was 1,5-AG ≥6.0 μg/mL.

Abbreviations: 1,5-AG, 1,5-anhydroglucitol; CI, confidence interval; HR, hazard ratio; TIR, time in range (3.9–10.0 mmol/L). ^*^*P* < 0.05, ^**^*P* < 0.01, ^***^*P* < 0.001

# **Supplementary Table 9. Incremental predictive value of adding serum 1,5-AG to Model 2 at different time points stratified by TIR**

| Group | Time point | C-statistic  (Model 2) | C-statistic  (Model 2 + 1,5-AG group) | *P* for change in C-statistic | Continuous NRI (95%CI) | IDI (95%CI) |
| --- | --- | --- | --- | --- | --- | --- |
| TIR >70% | 3-year | 0.790 | 0.791 | >0.05 | 0.047 (-0.114–0.230) | 0.004 (-0.004–0.021) |
|  | 5-year | 0.797 | 0.805 | >0.05 | 0.105 (0.001–0.229) | 0.008 (-0.002–0.025) |
|  | 7-year | 0.830 | 0.836 | >0.05 | 0.119 (0.042–0.207) | 0.012 (0.001–0.031) |
| TIR ≤70% | 3-year | 0.793 | 0.796 | >0.05 | 0.031 (-0.156–0.169) | 0.000 (-0.003–0.005) |
|  | 5-year | 0.812 | 0.814 | >0.05 | 0.020 (-0.137–0.147) | 0.001 (-0.001–0.007) |
|  | 7-year | 0.791 | 0.791 | >0.05 | -0.017 (-0.125–0.106) | 0.000 (-0.001–0.005) |

*Notes:* Model 2 included age, sex, diabetes duration, body mass index, systolic blood pressure, triglycerides, high-density lipoprotein cholesterol, low-density lipoprotein cholesterol, current smoking status, estimated glomerular filtration rate, history of cancer, history of cardiovascular disease, and the use of insulin, oral hypoglycemic drugs, antihypertensive drugs, lipid-lowering drugs and antiplatelet drugs.

Abbreviations: 1,5-AG, 1,5-anhydroglucitol; CI, confidence interval; IDI, integrated discrimination improvement; NRI, net reclassification improvement; TIR, time in range (3.9–10.0 mmol/L).

# **Supplementary Table 10. Associations of CGM-derived glycemic variability metrics with all-cause mortality in total**

|  | MAGE (mmol/L) | | CV (%) | | SD (mmol/L) | |
| --- | --- | --- | --- | --- | --- | --- |
| Group | < 6.1 | ≥ 6.1 | < 28.3 | ≥ 28.3 | < 2.6 | ≥ 2.6 |
| Number of participants | 2443 | 1234 | 2422 | 1255 | 2382 | 1295 |
| Number of deaths | 330 | 192 | 309 | 213 | 292 | 230 |
| Person-years | 17707.94 | 8696.74 | 17637.92 | 8766.76 | 17389.22 | 9015.47 |
| Incidence rate (per 1000 person-years) | 18.64 | 22.08 | 17.52 | 24.30 | 16.79 | 25.51 |
| HRs (95% CIs) |  |  |  |  |  |  |
| Unadjusted | 1.00 | 1.20 (1.00-1.43) ^*^ | 1.00 | 1.41 (1.18-1.68) ^***^ | 1.00 | 1.55 (1.30-1.84) ^***^ |
| Model 1 | 1.00 | 1.17 (0.98-1.40) | 1.00 | 1.24 (1.04-1.48) ^*^ | 1.00 | 1.37 (1.15-1.62) ^***^ |
| Model 2 | 1.00 | 1.11 (0.92-1.33) | 1.00 | 1.14 (0.95-1.36) | 1.00 | 1.12 (0.92-1.37) |

*Notes:* Model 1 included age and sex; Model 2 further included diabetes duration, body mass index, systolic blood pressure, triglycerides, high-density lipoprotein cholesterol, low-density lipoprotein cholesterol, current smoking status, estimated glomerular filtration rate, history of cancer, history of cardiovascular disease, and the use of insulin, oral hypoglycemic drugs, antihypertensive drugs, lipid-lowering drugs and antiplatelet drugs. For glycemic variability metrics, MAGE, CV, and SD were categorized into tertiles, with the lower two tertiles as the reference group. For the analysis of SD with all-cause mortality, model 2 was additionally adjusted for mean sensor glucose.

Abbreviations: CGM, continuous glucose monitoring; CI, confidence interval; CV, coefficient of variation; HR, hazard ratio; MAGE, mean amplitude of glycemic excursions; SD, standard deviation. ^*^*P* < 0.05, ^**^*P* < 0.01, ^***^*P* < 0.001
